# De Novo Deletion in the 12q24.23q24.31 Chromosomal Region Causing a Neurodevelopmental Syndrome in a Female Saudi Patient: A Case Report

**DOI:** 10.7759/cureus.78141

**Published:** 2025-01-28

**Authors:** Lina Bazeeb, Hanan A Aljedani, Manar S Alghamdi, Bayan Jamjoom, Aiman M Shawli

**Affiliations:** 1 College of Medicine, King Saud Bin Abdulaziz University for Health Sciences, Jeddah, SAU; 2 Medicine and Surgery, King Abdullah International Medical Research Center, Jeddah, SAU; 3 Pediatrics, King Abdullah Specialist Children’s Hospital, Jeddah, SAU; 4 Genetics and Precision Medicine, King Abdullah Specialist Children’s Hospital, Jeddah, SAU; 5 General Pediatrics and Pediatric Genetics, King Abdullah Specialist Children’s Hospital, Jeddah, SAU

**Keywords:** 12q24.23q24.31 chromosomal region deletion, developmental delays, epilepsy, intellectual difficulties, neurodevelopmental abnormalities

## Abstract

A substantial loss in the 12q24.23q24.31 area has been associated with neurodevelopmental abnormalities, intellectual difficulties, and developmental delays. There are several chromosomal deletion syndromes worldwide, each with its own set of features. However, the rarity of this deletion in 12q24.23q24.31 presents an opportunity to expand the existing knowledge on this topic.

Our case involved a seven-year-old girl with no history of consanguinity, who was discovered to have a 6.6 Mb deletion in the 12q24.23q24.31 region. She was diagnosed with refractory epilepsy, spasticity in all limbs, and global developmental delay with intellectual disabilities. She was able to do basic movements with assistance, identify familiar people, and respond to simple instructions. Some significant physical traits included widely separated eyes, a small nasal tip, and congenital heart defects, such as tricuspid atresia and a single ventricle heart. She also demonstrated clubbing in her fingers and toes, as well as toe overlapping. The purpose of this case report is to contribute to our understanding of deletions in the 12q24.23q24.31 chromosomal region and their clinical implications.

## Introduction

When chromosomal segments are lost, chromosomal deletion syndromes can result, which can cause severe prenatal abnormalities, as well as intellectual and physical difficulties. They tend to be identified postnatally based on clinical symptoms and confirmed using karyotyping for more extensive deletions, or complicated cytogenetic methods like fluorescence in situ hybridization or microarray analysis for smaller deletions [1].

A unique neurodevelopmental phenotype, which includes behavioral abnormalities, intellectual impairment, and developmental delay, has been associated with deletions in the 12q24.23q24.31 region [2]. This case makes a unique addition to our understanding of 12q24 deletions since it showed a case of a heterozygous deletion in this specific genomic region. This case encompasses a broader range of abnormalities, including global developmental delay, refractory epilepsy, and severe congenital heart disease.

Furthermore, the association with the *HNF1A* gene, known for its link to maturity-onset diabetes of the young (MODY) type III, introduces a new perspective, highlighting the potential for early endocrinological diagnosis and management. This combination of observations, together with the detection of this particular loss, emphasizes the case's rarity and relevance in increasing our phenotypic and molecular understanding of 12q24 deletions. This case report presents a seven-year-old Saudi girl with a complex clinical presentation, including global developmental delay, refractory epilepsy, and complex congenital heart disease. The condition is brought on by a deletion in the 12q24.23q24.31 chromosome, which also increases the risk of developing MODY type III.

## Case presentation

Our patient is a seven-year-old Saudi girl who presented for genetic evaluation. She was born full-term to non-consanguineous parents who have no medical concerns. She has two younger sisters and one paternal half-brother, all of whom have no medical or genetic concerns. The family history was unremarkable for any other medical conditions. Her pregnancy was complicated by intrauterine growth restriction (IUGR); her birth weight was 2 kg (less than the third percentile), her length was 45 cm (less than the third percentile), and her occipitofrontal circumference was 31 cm (less than the third percentile). Shortly after birth, she was admitted to the Neonatal Intensive Care Unit (NICU) as a case of complex congenital heart disease, including tricuspid atresia, single ventricle heart, hypoplastic right ventricle, and ventricular septal defect (VSD), and has undergone patent ductus arteriosus ligation and a right-sided Glenn procedure. She also has refractory epilepsy, which is partially controlled with medications (clobazam 14 mg BID, levetiracetam 450 mg BID, valproic acid 250 mg BID). However, she continues to experience intermittent sleep-related spasms and generalized tonic-clonic seizures, which are being managed by pediatric neurology. There was an early global delay in developmental milestones. She achieved head control at 24 months and was able to sit independently by 48 months.

Currently, at seven years old, the patient’s height is 100 cm, weight is 13 kg, and head circumference is 46 cm - all below the third percentile, indicating failure to thrive. Developmentally, she can sit and stand with support, roll over in bed, grasp and mouth objects, follow simple routine commands, and recognize her name as well as familiar faces. Physical features were significant for hypertelorism, a depressed nasal bridge, and a small, peaked nasal tip. Additionally, she shows spasticity in all limbs, clubbing of the fingers and toes, and bilateral overlapping of the third and fourth toes.

**Figure 1:**
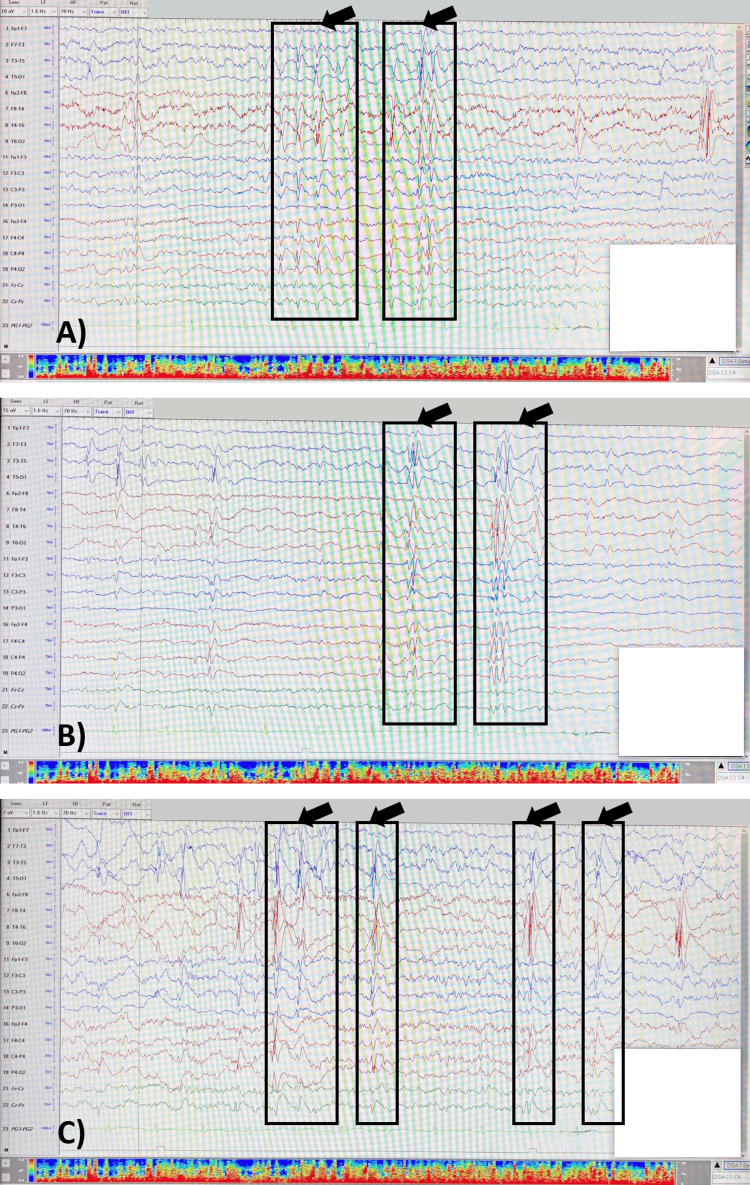
Electroencephalogram findings (A) Sensitivity 10; (B) Sensitivity 15; (C) Sensitivity 7

**Figure 2:**
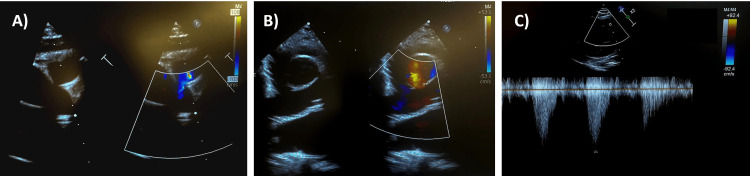
Cardiac echocardiography (A) Ventricular septal defect; (B) Tricuspid atresia; (C) Glenn shunt

The patient’s vital signs and routine laboratory results, including hemoglobin A1C, are within normal limits. Electroencephalogram findings reveal active, generalized epileptiform abnormalities, aligning with her condition (Figure 1). Echocardiography shows an unobstructed Glenn shunt with good flow in the right and left pulmonary arteries, a good-sized atrial communication, and an unobstructed aortic arch (Figure 2). Ventricular systolic function is preserved, and there is no evidence of obstruction in the cavopulmonary connection, indicating stable postoperative hemodynamics. The patient is actively following physical and occupational therapy for her global developmental delay, resulting in significant improvements in motor activities and sensory integration. She continues to receive care from a multidisciplinary team to manage and support her condition effectively. The patient was referred to pediatric genetics from cardiology due to complex heart disease, and whole exome sequencing (WES) was done. The results revealed a heterozygous deletion of approximately 6.6 Mb within the 12q24.23q24.31 chromosomal region (copy number state: 1), classified as likely pathogenic. Familial segregation analysis suggests that the variant is de novo, although germline mosaicism in one of the parents cannot be ruled out. This finding was confirmed by chromosomal microarray (CMA) analysis performed on the family trio as an internal control.

The detected deletion includes the *HNF1A* gene, linked to MODY type III (OMIM: 600496). HNF1A-MODY typically leads to diabetes onset in late adolescence or early adulthood. Given the patient’s risk of developing MODY, the patient was referred to endocrinology for screening, and the test results were normal.

The library was prepared using the DNA Capture Probe Kit and sequenced on the Illumina platform. A confirmatory analysis was then performed using CMA with the Infinium Global Diversity Array Kit with cytogenetics. The genes that were involved in this mutation were 122 genes, as follows: *LINC02423, LINC02439, SRRM4, LOC105370024, HSPB8, LINC00934, CCDC60, TMEM233, PRKAB1, MIR1178, CIT, BICDL1, RAB35, MIR4498, GCN1, RPLP0, PXN-AS1, PXN, RNU4-2, RNU4-1, SIRT4, PLA2G1B, MSI1, COX6A1, TRIAP1, GATC, SRSF9, DYNL1, NRAV, COQ5, RNF10, POPS, CABP1, MLEC, UNC119B, MIR4700, ACADS, SPPL3, XLOC_00991, HNF1A-AS1, HNF1A, C12orf13, OASL, P2RX7, P2RX4, CAMKK2, ANAPC5, RNF34, MIR7107, KDM2B, ORAI1, MORN3, TMEM120B, RHOF, LINC01089, SETD1B, HPD, PSMD9, CFAF251, BCL7A, LOC105005691, MLIP, L3, LRRC43, B3GNT4, DIABLO, LOC101593348, VPS33A, CLIP1, CLIP1-AS, ZCCHC8, RSRG2, KNTC1, MIR9902-2, HCAR2, MIR9902-1, HCAR3, HCAR1, DENR, CCDC62, HIP1R, VPS37B, ABCB9, OGFOD2, ARL6IP4, MIR4304, PITPNM2, PITPNM2-AS1, MPHOSPH9, C12orf65, CDK2AP1, SBNO1, MIR8072, SBNO1-AS1, KMT5A, RILPL2, SNRNP35, RILPL1, MIR3908, TMED2-DT, TMED2, DDX55, SNORA98, EIF2B1, GTF2H3, TCTN2, ATP6V0A2, DNAH10, CCDC92, ZNF664, ZNF664-RFLNA, RFLNA, MIR6880, NCOR2, SCARB1, UBC, MIR5188, DHX37, BR13BP, THRIL, AACS, TMEM132B.*

## Discussion

This is a case of a seven-year-old girl with refractory epilepsy and complicated congenital heart disease, including tricuspid atresia and a single ventricle heart. She also has global developmental delay, intellectual disabilities, and restricted motor capabilities. Deletions in the 12q24.23q24.31 region were discovered when performing WES, with a size of 6.6 Mb, de novo. This report makes a distinct contribution to the literature on 12 chromosomal deletions by presenting a patient with a mix of complicated conditions that provide a larger clinical spectrum associated with this genetic abnormality.

Our patient has various distinguishing features compared to previous research findings. There was no history of consanguinity, and the case had a 6.6 Mb deletion, which falls in the middle of previously recorded deletions ranging from 1.6 Mb to 9.2 Mb (Table 1). Neurologically, our patient had distinct characteristics, including refractory epilepsy, a condition seldom reported in similar cases and only mentioned in one prior paper [2]. Spasticity was consistent with previous descriptions, and developmental delays and intellectual deficits were also present, as stated in every known case. Notably, our patient displayed an exceptional capacity to understand simple routines and orders, as well as identify her name and familiar faces, highlighting her cognitive talents despite global developmental impairments (Table 1).

**Table 1:** Comparison between our study's manifestations and other manifestations mentioned in the literature M: Male; F: Female; RE: Refractory Epilepsy; SRS-GS: Sleep-Related Spasms and Generalized Seizures; SL: Spasticity in Limbs; BMS: Basic Motor Skills (Sit, Stand with Support, Roll, Hold Objects); UCR: Understands Commands, Recognizes Names/Faces; SQ: Spastic Quadriplegia; SHBTR: Spastic Hypertonus and Brisk Tendon Reflexes; UWIS: Unable to Walk Independently or Speak; HC: Hydrocephalus; HPT: High Pain Threshold; HFSB: Hyperactivity with Food-Seeking Behavior; ADHD: Attention Deficit Hyperactivity Disorder; OCT: Obsessive-Compulsive Tendencies; SII: Self-Inflicted Injury; GDD: Global Developmental Delay; ID: Intellectual Disability; PDM: Poor Developmental Milestones; FTT: Failure to Thrive; TA: Tricuspid Atresia; SVH: Single Ventricle Heart; HRV: Hypoplastic Right Ventricle; VSD: Ventricular Septal Defect; PFO: Persistent Open Foramen Ovale; MMR: Mild Mitral Regurgitation

	Present case	​​Palumbo et al. (2015) [2]	Lin et al. (2020) ​​[3]	​​Al-Zahrani et al. (2011) [4]	Niyazov et al. (2007) ​​[5]	
					Patient 1	Patient 2
Age, gender	7y, F	11y, F	12y, M	10y, M	8y, M	12y, M
Consanguinity	Nonconsanguineous parents	Nonconsanguineous parents	First double cousin	First cousins	-	
Size of deletion	6.6 Mb 12q24.23q24.31	1.66 Mb 12q24.31	9 Mb del (12) (q24.31-q24.33)	9.2 Mb on chromosome 12q24.31-q24.33	1.6 Mb	4.5 Mb
Neurological - behavioral	RE, SRS-GS, SL, BMS, UCR	Seizures, SQ, stereotypies (repetitive movements), anxiety	SHBTR, UWIS, HC	-	HPT, HFSB, ADHD	HPT, OCT, SII
Developmental	GDD, ID	GDD, ID	Early GDD, developmental retardation, FTT	PDM, GDD, severe growth retardation, limited speech	ID, delayed milestones, language development delay	ID, delayed milestones and moderate learning disabilities
Craniofacial features	Hypertelorism, depressed nasal bridge; Small, peaked nasal tip	Downslanted palpebral, fissures, broad nasal base with high nasal root; high palate with overcrowded teeth and macroglossia; full cheeks, large narrow ears with a thick helix, full and everted lower lip	Slight coarse facial features; short nose with anteverted nares, smooth philtrum; narrow palate with thick gums	Microcephaly, micrognathia; long philtrum, small ears with deformity	Anterior hair whorl; absence of significant dysmorphic facial features	Epicanthal folds; small ears
Cardiac (congenital)	TA, SVH, HRV, VSD	PFO, MMR	-	-	-	-
Genital (urogenital)	-	-	Undescended testicles	Micropenis, undescended testes, abnormal genital development	-	Left undescended testicle
Musculoskeletal	Clubbing of the fingers and toes; bilateral overlapping of the third and fourth toes	Mild tapering of fingers; hypoplastic nails with proximal implantation of the 4th metacarpal bone	Clinodactyly, polydactyly	Bent elbows, kyphoscoliosis	Brachydactyly, mild clinodactyly	-
Endocrine	-	Neonatal hypoglycemia and hypocalcemia	-	Suspicion of panhypopituitarism, but thyroid and IGF-1 levels were inconclusive	Obesity: BMI >97th percentile	Obesity: BMI >97th percentile

The smallest deletion discovered, measuring 1.6 Mb, was remarkable for the lack of substantial dysmorphic facial characteristics, as opposed to previously reported instances, which frequently had noticeable dysmorphic traits. As shown in Table 1, our patient had unique cardiac defects, such as tricuspid atresia, a single ventricle heart, a hypoplastic right ventricle, and a VSD. These cardiac manifestations are novel and have not been described in any previously reported cases. Furthermore, unlike previous studies, our patient showed no substantial genital or endocrinological abnormalities. Our patient's musculoskeletal abnormalities included clubbing and overlapping of the third and fourth toes. This presentation contrasts with previous research, which has reported kyphoscoliosis, clinodactyly, and polydactyly (Table 1).

In this case, the loss had a major influence on a large number of genes, with 122 genes identified. To our knowledge, the majority of these genes have not been thoroughly studied in the literature. One prominent exception is the *ACADS* gene, which has been associated with neurodevelopmental problems. This gene was previously identified in research by Lin et al., stressing its possible relevance to the phenotype [3]. It is worth mentioning, however, that the patient reported in Lin et al.'s research had autism spectrum disorder, whereas our patient in this case had no symptoms consistent with autism spectrum disorder. This difference emphasizes the heterogeneity in clinical manifestations associated with comparable genetic deletions, highlighting the complexities of genotype-phenotype connections. *P2RX2*, an important gene that was not affected in our case, has been mentioned in three different studies. This gene is significantly related to neurological disorders and behavioral difficulties [3-5].

In this case report, we found an essential gene that was impacted by this deletion: *HNF1A*. This gene plays a role in regulating pancreatic β-cell development, maintenance, and glucose-induced insulin release through a transcription factor network. Mutations in *HNF1A* can cause a variety of protein malfunctions, ranging from severe loss-of-function (LOF) variants connected to highly penetrant MODY to milder LOF variants with lower penetrance, yet increasing the risk of type 2 diabetes in the population by up to fivefold [6]. This suggests the possibility of early endocrinological intervention, emphasizing the significance of a complete, multidisciplinary approach to the treatment of patients with 12q24 deletions who have this gene mutation.

Chromosome deletions play a crucial role in the beginning and progression of neurodevelopmental disorders. One study provides valuable insights into the intricate connection between these deletions and neurodevelopmental abnormalities, demonstrating that such deletions frequently result in disruption of the gene clusters that are critical for brain development, affecting genes involved in circulation, immunological response, and neuronal growth, leading to severe neurological deficits [7]. For illustration, the particular manifestations of chromosomal deletions vary depending on the chromosome in consideration; however, they usually share similarities in presentations as well as some differences. For instance, chromosome 12 deletions are linked to epilepsy, autism, and intellectual disabilities, usually with normal MRI findings. On the other hand, the 15q11.2 deletion is linked to epilepsy, intellectual disability, and schizophrenia, as well as structural brain abnormalities like diminished cortical surface area, increased cortical thickness, and white matter abnormalities [8]. Additionally, larger deletions on chromosome 17p13.3 result in Miller-Dieker syndrome, whereas smaller deletions induce lissencephaly, intellectual impairments, and developmental delays [9]. This diversity in manifestations is caused by two main mechanisms: firstly, structural changes affecting the shape and connection of neurons; secondly, genomic instability, where deletions affect gene regulation and protein-coding processes, exacerbating neurodevelopmental abnormalities [8,10].

## Conclusions

Chromosome deletions are associated with neurodevelopmental abnormalities such as autism spectrum disorder, intellectual difficulties, epilepsy, and other disorders, and they can present with a variety of manifestations. This case of a seven-year-old female presented with a unique set of symptoms, including refractory epilepsy and rare cardiac anomalies, highlighting undocumented traits in the literature. These findings emphasize the importance of further research into this specific chromosomal deletion in order to improve our understanding and expand the existing body of knowledge, which will, in turn, improve diagnostic and therapeutic approaches for patients.

## References

[1] (2024). Chromosomal deletion syndromes. https://www.msdmanuals.com/professional/pediatrics/chromosome-and-gene-abnormalities/chromosomal-deletion-syndromes.

[2] Palumbo O, Palumbo P, Delvecchio M (2015). Microdeletion of 12q24.31: report of a girl with intellectual disability, stereotypies, seizures and facial dysmorphisms. Am J Med Genet A.

[3] Lin J, Souza-Lin GR, Antunes FC, Wessler LB, Streck EL, Gonçalves CL (2020). Autism associated with 12q (12q24.31-q24.33) deletion: further report of an exceedingly rare disorder. Einstein (Sao Paulo).

[4] Al-Zahrani J, Al-Dosari N, Abudheim N (2011). Chromosome 12q24.31-q24.33 deletion causes multiple dysmorphic features and developmental delay: first mosaic patient and overview of the phenotype related to 12q24qter defects. Mol Cytogenet.

[5] Niyazov DM, Nawaz Z, Justice AN, Toriello HV, Martin CL, Adam MP (2007). Genotype/phenotype correlations in two patients with 12q subtelomere deletions. Am J Med Genet A.

[6] Kavitha B, Ranganathan S, Gopi S, Vetrivel U, Hemavathy N, Mohan V, Radha V (2023). Molecular characterization and re-interpretation of HNF1A variants identified in Indian MODY subjects towards precision medicine. Front Endocrinol (Lausanne).

[7] Friedenson B (2019). A genome model to explain major features of neurodevelopmental disorders in newborns. Biomed Inform Insights.

[8] Habela CW, Liu S, Taga A (2024). Altered development and network connectivity in a human neuronal model of 15q11.2 deletion-related neurodevelopmental disorders (Preprint). bioRxiv.

[9] Blazejewski SM, Bennison SA, Smith TH, Toyo-Oka K (2018). Neurodevelopmental genetic diseases associated with microdeletions and microduplications of chromosome 17p13.3. Front Genet.

[10] Cao Y, Luk HM, Zhang Y (2022). Investigation of chromosomal structural abnormalities in patients with undiagnosed neurodevelopmental disorders. Front Genet.

